# Different Rescue Approaches of Migrated Woven Endobridge (WEB) Devices: an Animal Study

**DOI:** 10.1007/s00062-020-00893-3

**Published:** 2020-03-12

**Authors:** Andreas Simgen, Michael Kettner, Philipp Dietrich, Toshiki Tomori, Ruben Mühl-Benninghaus, Pervinder Bhogal, Matthias W. Laschke, Michael D. Menger, Wolfgang Reith, Umut Yilmaz

**Affiliations:** 1 Department of Neuroradiology, Saarland University Hospital, Kirrbergerstraße 1, 66424 Homburg/Saar, Germany; 2 Institute for Clinical & Experimental Surgery, Saarland University, Homburg/Saar, Germany; 3 Department of Interventional Neuroradiology, The Royal London Hospital, Whitechapel Road, E1 1BB London, UK

**Keywords:** JET 7, SOFIA Plus, Alligator retrieval device, Microsnare

## Abstract

**Purpose:**

Treatment of wide-necked intracranial aneurysms using the Woven Endobridge (WEB) device has become broadly accepted. Feared complications with the potential of increased poor clinical outcome include dislocations and migration of the device. This study was carried out to determine the effectiveness of a variety of different strategies to rescue migrated WEB devices.

**Methods:**

In a porcine model, WEB devices of different sizes (SL [single layer] 3.5 × 2mm and SL 4.0 × 3 mm, SL 8 × 5 mm and SLS 8 mm [single layer spherical]) were placed into both the subclavian and axillary arteries. A total of 32 rescue maneuvers (8 per rescue device) were performed. Small WEBs were rescued using reperfusion catheters (RC) (SOFIA Plus and JET 7), larger WEBs were rescued using dedicated rescue devices (Microsnare and Alligator). Rescue rates, times, attempts and complications were assessed.

**Results:**

Rescue attempts of migrated WEBs were successful in all cases (100%). Rescue time (*p* = 0.421) and attempts (*p* = 0.619) of small WEBs using RCs were comparable without significant differences. Aspiration alone was not successful for larger WEBs. Rescue of larger WEBs was slightly faster (122.75 ± 41.15 s vs. 137.50 ± 54.46 s) with fewer attempts (1 vs. 1.37) when using the Microsnare compared to the Alligator device. Complications such as entrapment of the WEB in the RCs, vasospasm, perforation, or dissection were not observed.

**Conclusion:**

Rescue of migrated WEB devices is a feasible and effective method and 100% successful rescue rates and appropriate rescue times can be achieved for small WEBs using RCs and for larger WEBs using dedicated rescue devices (Microsnare and Alligator).

## Introduction

The Woven Endobridge (WEB) device (MicroVention, Tustin, CA, USA) entered the market in 2011 and was designed to treat wide-necked intracranial aneurysms without the need for neck supporting devices (e.g. balloons or stents) or dual antiplatelet therapy [1]. Many studies have shown promising results in the treatment of wide-necked intracranial aneurysms with high rates of adequate occlusion and low morbidity and mortality rates [2, 3]. With the introduction of the new generation (low-profile) WEB-17 device even small (<5 mm) and distal aneurysms (M2–3 segments) can be treated safely and efficiently [4, 5]. Even in the setting of a ruptured aneurysm WEB devices have shown acceptable results concerning occlusion rates and the risk of rebleeding [6–8]. Despite the device being intrasaccular in nature, thromboembolic complications can occur and particularly in wide-necked aneurysms given the increased surface area in contact with the blood [9]. Dislocation and migration of WEBs are isolated complications that have only been reported twice [10, 11]. Many different endovascular bail-out techniques, using a variety of strategies and equipment have been described for rescuing other devices, such as coils and stents [12–17]. A systematic rescue approach of migrated WEBs has not been investigated so far.

The purpose of this study was to assess the feasibility and effectiveness of rescuing migrated WEBs using a variety of different reperfusion catheters (RC) and dedicated rescue devices.

## Material and Methods

### Animal Care

The governmental protection committee approved the animal experiments. The experiments were performed in accordance with the European legislation on the protection of animals (Directive 2010/63/EU) and the National Institute for Health (NIH) guidelines on the care and use of laboratory animals (NIH publication #85-23 Rev. 1985). They were performed in 2 female Swabian Hall pigs (body weight 40–50 kg), as previously described in detail [15].

### WEB Device

**Fig. 1:**
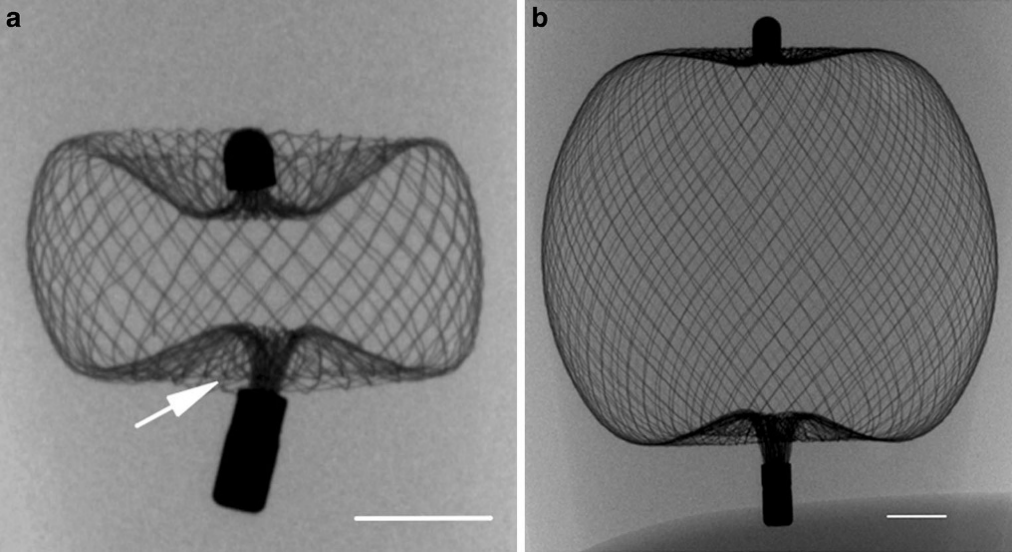
Microcomputed tomography (CT) fluoroscopy of the rescued WEB devices SL 3.5 × 2 mm (**a**) and SLS 8 (**b**). The white arrow indicates the slight deformation at the proximal marker. Scale bar = 1 mm

The WEB device is an electrolytically detachable braided basket and has between 114 and 216 nitinol wires. Distal and proximal it holds platinum markers. The area of the proximal marker also serves as the detachment zone. There are two differently shaped devices (WEB SL and WEB SLS, spherical) available in a variety of different sizes (Fig. 1a,b; [1, 18]).

### Reperfusion Catheters

#### SOFIA Plus

The SOFIA Plus (Microvention) catheter was FDA and CE market approved in 2015. It has a hybrid braid-coil design and is available in two lengths (125 cm and 131 cm). The outer diameter measures 6F, the inner luminal diameter 0.070 in. [19].

#### JET 7

The JET 7 (Penumbra, Alameda, CA, USA) is the company’s 7th generation RC. It received FDA approval in 2018 and was just recently granted a CE mark. The catheter features 20 transitions from the proximal shaft to the distal tip. It possesses a progressive distal coil wind for more flexibility and Quad-Wire technology in the proximal shaft for enhanced pushability. The outer diameter measures 6F and the inner lumen diameter 0.072 in. [20].

### Dedicated Rescue Devices

#### Microsnare

Amplatz GooseNeck Microsnare (Medtronic/eV3, Irvine, CA, USA) is made of nitinol and possesses a 90° angled loop. It is available with a diameter of 4 mm and 7 mm and a length of 175 cm.

### Alligator Retrieval Device (ARD)

The ARD (Medtronic/eV3) possesses 4 hock-like tentacles attached to a 0.0016 in. stainless steel wire. The device is available with a wing spread of 3 mm and 175 cm.

### Intervention

The endovascular procedures were performed by two neurointerventionalists (U.Y. 10 years of interventional experience, A.S. 6 years of interventional experience). All interventions were conducted under fluoroscopy using a monoplane angiographic system (Ziehm Vision imaging, Nuremberg, Germany). Ultravist 370 (iopromide; Bayer Schering Pharma, Berlin, Germany) was used as a contrast agent. Endovascular procedures were performed after an intravenous bolus injection of heparin (5000 IU, Braun, Melsungen, Germany) and nimodipine (2 mg, Carinopharm GmbH, Elze, Germany).

### Positioning of the WEB Device

**Fig. 2:**
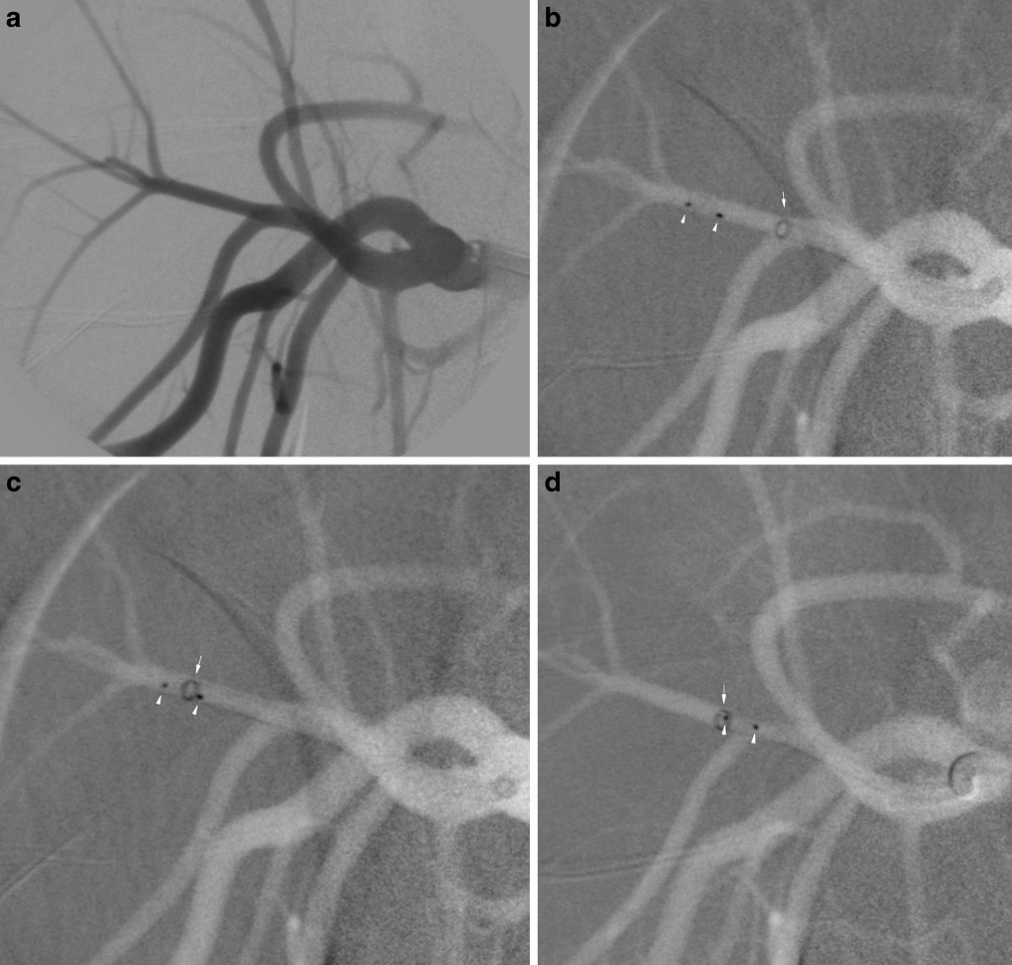
**a** Digital subtraction angiography (DSA) of the right subclavian artery, axillary arteries and their branches. **b** Roadmap image of the migrated WEB device (SL 3.5 × 2 mm) with its distal and proximal radiopaque marker (*white arrowheads*) and the JET 7 (*white arrow*). **c** Advancement of the JET 7 (*white arrow*) until the proximal marker (*white arrowhead*) was inside the JET 7. **d** After suction had been applied, the WEB device (*white arrowheads*) was fully aspirated into the JET 7 and the catheter was withdrawn (*white arrow*)

Supported by a 0.035 in. guide wire (Terumo, Tokyo, Japan) and a 5F vertebral catheter (Cordis Endovascular, Santa Clara, CA, USA) a long sheath (6F Neuron MAX Penumbra Inc., Alameda, CA, USA) was placed in the proximal subclavian artery (Fig. 2a). The target vessels were reached with the aid of a Traxcess 0.014-inch microwire (Microvention) and either a VIA-17 or VIA-27 microcatheter (Microvention), depending on the size of the WEB device. The first WEB device was placed and electrolytically detached. Following detachment, the final position of the device was left to the flow of blood and not manipulated any further. In all cases the WEBs aligned perpendicular to the vessel.

Due to the limited experimental supply, the WEBs were reused as far as no damage could be detected after macroscopic inspection. Therefore, a 4-mm Microsnare (which was previously inserted in a retrograde manner into the catheter) was attached to the proximal marker and the WEB was gently pulled back into the microcatheter. Smaller WEBs were pulled back into the VIA-27 microcatheter and larger WEBs had to be pulled into a 4MAX RC (Penumbra). Subsequently, positioning of the WEBs was manually achieved by gently pushing the Microsnare while simultaneously retracting the catheter until the WEB device was deployed.

### Rescue Approaches of the WEB Device

Regardless the size of the WEB, the first rescue approach was direct aspiration (flow-controlled technique without microcatheter and microwire) using the selected RC (SOFIA Plus or JET 7). The aspiration approach was similar to the ADAPT technique (A Direct Aspiration first Pass Technique) commonly used in the treatment of ischemic stroke [21]. Upon reaching the WEB device, the RC was gently advanced until the proximal marker of the WEB was inside the catheter (proximal to the distal marker of the RC) (Fig. 2b,c). After this had been achieved, aspiration was applied in case of the SOFIA using a 60 mL VacLok syringe (Merit Medical, South Jordan, UT, USA) and in the case of the JET 7 using an aspiration pump (Penumbra). If the WEB device was completely aspirated, the RC was withdrawn (Fig. 2d). If the WEB device was only partially ingested into the RC, the catheter was advanced in order to achieve more ingestion. If aspiration failed, rescue of the WEBs was performed via the same RC using a Microsnare and the ARD. The ARD was introduced into the VIA-27 microcatheter in order to capture the WEB with the proximal marker of the WEB being the target. The Microsnare was inserted into the RC along with its delivery snare catheter. The rescue approach with the snare was to rope the WEB device and subsequently trap it with the snare catheter. Under continuous tension of the Microsnare and ARD the WEBs were extracted through the RCs.

### Angiographic Evaluation

With each rescue approach it was intended to rescue 8 positioned WEBs at different positions within the arteries. Rescue was considered successful if the WEB device was removed from the animal. After each rescue maneuver, digital subtraction angiography (DSA) was performed to evaluate vessel complications. The following parameters were assessed:

1. Rescue rates for each approach.
2. Rescue time, defined as time beginning from navigation to the WEB until successful extraction.
3. Number and total rescue attempts.
4. Complications: vasospasm, perforation, dissection, entrapment at RC.

### Statistical Analysis

Continuous variables are expressed as means and standard deviations. Categorical variables are presented as absolute and relative frequencies, unless otherwise stated. Fisher’s exact tests were performed for the comparison of categorical variables between the groups. Continuous variables were tested for normal distribution. To study differences between the groups, Student’s t‑test was applied. Statistical significance was accepted at a two-sided *p* value of <0.05. All data analyses were performed using SPSS Statistics 22™ (IBM, Chicago, IL, USA).

## Results

### Vessel and WEB Sizes

Using the aforementioned rescue approaches a total of 32 rescue maneuvers were performed in 2 pigs. With each RC and dedicated rescue device (SOFIA Plus, JET 7, Microsnare and ARD) a total of 8 maneuvers was performed. For this purpose, the target vessels were the subclavian and axillary arteries with a mean diameter of 3.72 ± 1.32 mm (animal 1) and 3.65 ± 1.59 mm (animal 2). A total of 8 WEBs of different sizes was used (SL 3.5 × 2 mm, *n* = 2, SL 4.0 × 3 mm, *n* = 2, SL 8 × 5 mm, *n* = 2 and SLS 8 mm, *n* = 2).

### Rescue Rate, Time and Attempts

Successful rescue was achieved in all 32 cases, corresponding to a rescue rate of 100%. The results of the different rescue approaches are presented in detail in Table 1. Rescuing smaller WEBs with the applied RC was achieved with an overall rescue time of <1–2 min. In 3 cases during aspiration with the SOFIA and 2 cases with the JET 7, the first aspiration attempt failed since the WEBs were lost during retraction of the RC. In these cases the ADAPT technique was immediately repeated. No more than two aspiration attempts were required. In most cases the WEB device was ingested completely and remained within the distal portion of the RC. In only a few cases, complete aspiration through the RC was observed. *Slightly reduced rescue times and attempts were experienced* with the JET 7 *in comparison to the* SOFIA Plus; however, this difference was not statistically significant (*p* = 0.421/*p* = 0.619).

**Table 1:** Overview of the results comparing the different rescue approaches presented as means ± standard deviations.

Rescue devices	WEB Devices	Rescue rates [%]	Time of rescue [s]	Attempts, mean [*n*]	Perforation [*n*]	Dissection [*n*]	Vasospasm [*n*]	Entrapment at RC [*n*]
SOFIA Plus	SL 3.5 × 2/4 × 3	100	69.62 ± 37.65	1.37	0	0	0	0
JET 7	SL 3.5 × 2/4 × 3	100	54.25 ± 36.51	1.25	0	0	0	0
*p*-value	–	–	0.421	0.619	–	–	–	–
Microsnare	SL 8 × 5/SLS 8	100	122.75 ± 41.15	1	0	0	0	0
Alligator	SL 8 × 5/SLS 8	100	137.50 ± 54.46	1.37	0	0	0	0
*p*-value	–	–	0.551	0.060	–	–	–	–

*RC* reperfusion catheter

**Fig. 3:**
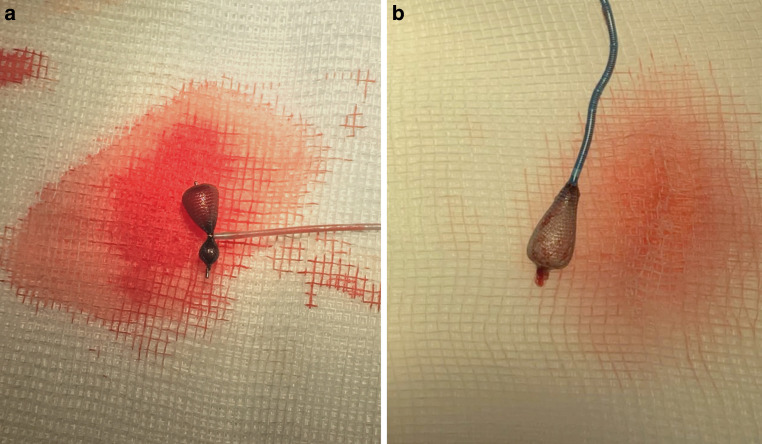
**a** Rescue of the WEB device (SLS 8) using the Microsnare leading to an hourglass configuration. **b** Rescue of the WEB device (SLS 8) using the ARD at the proximal marker with partially resheated WEB device within the VIA-27 microcatheter

The rescue of larger WEBs by aspiration alone was not successful in any case. Therefore, the rescue approach was escalated using dedicated rescue devices. With the Microsnare, rescue was possible at the first attempt with a rescue time of 1–3 min. In all cases, the Microsnare could be placed over and around the first half of the WEB device, leading to an hourglass configuration once the WEB was trapped (Fig. 3a). Retraction through the RC was achievable with minimal resistance. Using the ARD, a rescue time of 1–4 min was achieved. In most cases, capture of the WEB device was achieved at the proximal marker or its surrounding area. In 2 cases, it was possible to fully capture the proximal marker and a partial retrieval of the WEB device back into the microcatheter (Fig. 3b). In 3 cases, a second rescue attempt had to be performed because the WEB was lost once it was about to be pulled into the RC. Comparing both dedicated rescue devices shorter rescue times and fewer attempts were observed when using the Microsnare although this difference was not statistically significant (*p* = 0.551/*p* = 0.060).

### Complications

 No cases of vasospasm, perforation or dissection were observed. Neither entrapment of the WEB device at the reperfusion catheter was seen.

### Micro-CT of the Rescued WEB Devices

Acquisition of micro-CT images of the used WEBs was performed as previously described in detail [22]. Although the WEBs were subjected to a high mechanical force as a result of their multiple use, their structure remained unchanged (Fig. 1a,b). Only a slight deformation of the smaller WEBs at the wires merging into the proximal marker was observed (Fig. 1a). At this location, the Microsnare was applied to pull the WEB device back into the catheter in order to use it multiple times.

## Discussion

Treatment of wide-necked intracranial aneurysms using WEBs is still a relatively new approach compared to other endovascular procedures but has evolved into a safe and effective method. Lv et al. reported in a systematic review a technical success rate of 97% and adequate occlusion rates of 80% within the first year of observation. The most commonly reported complications were thromboembolic (8%) and bleeding (2%) events with an overall morbidity rate of 3% [9]. Complications, such as dislocation and migration seem to be very rare and have not yet been frequently described in the literature. Such complications have neither been described in the larger WEB trials (WEB-IT, WEBCAST) nor in a recent meta-analysis [2, 3, 23].

König et al. [10] were the first who described the only case of a dislocated and migrated WEB device during treatment of a wide-necked aneurysm of the right ICA bifurcation. After successful detachment of the delivery wire during withdrawal of the microcatheter, the WEB device dislocated completely and migrated into the MCA bifurcation. Rescue was performed using an ARD [10]. John et al. [11] published one more case of a dislocated WEB device during treatment of a MCA bifurcation aneurysm. They described an incomplete detachment of the WEB device used causing a partial dislocation out of the aneurysm during retrieval of the delivery wire. The WEB device was not rescued, instead they used a microcatheter to push it completely back into the aneurysm [11].

To the best of our knowledge a systematic rescue approach of migrated WEBs has not been investigated so far. The purpose of this study was to assess the feasibility and effectiveness of rescuing migrated WEBs of different sizes using a variety of RCs and dedicated rescue devices.

Ever since the ADAPT technique was introduced, the continuous development of large-bore catheters has made it a very effective and timesaving method in the treatment of large vessel occlusions [21, 24]. The SOFIA Plus was one of the first large-bore catheters available and has shown good results as a first-line aspiration catheter for endovascular stroke treatment [19]. Of the RCs currently available on the market, the JET 7 possesses the largest inner lumen at 0.072 inches. Studies of the JET 7 performance in the treatment of ischemic stroke are currently being conducted. Zaidat et al. have demonstrated first experiences (oral presentation at the 15th Congress of World Federation of Interventional and Therapeutic Neurology 2019) in an interim analysis of 114 patients and have shown higher FPE and faster revascularization times compared to non-JET catheters [25]. In this study, the SOFIA Plus as well as the JET 7 were able to achieve a successful rescue of smaller WEBs in 100% (16 of 16 cases). A slightly reduced rescue time and fewer attempts were observed using the JET 7, which could be related to the larger inner diameter compared to the SOFIA Plus; however, this difference was not statistically significant. In our experience, a second rescue attempt was necessary whenever the WEB device was not fully aspirated within the RC. Thus, advancing the RC under continuous aspiration until the WEB device is completely inside the RC is recommended in order to achieve a successful rescue with the first attempt. Because of the size of the applied RC, aspiration beyond vessel sizes of the M2 and P1 segments in humans will be limited.

Dedicated rescue devices, such as Microsnares and the ARD, have been available for some time and have even been used for mechanical thrombectomy [26, 27] prior to the era of stent-retriever thrombectomy. The most experiences using Microsnares and the ARD have been reported in the rescue of neurovascular devices, such as coils [12–15] and stents [16, 17, 28]. In the present study, the Microsnare as well as the ARD were able to achieve a successful rescue of larger WEBs in 100% (16 of 16 cases). Unfortunately, aspiration alone of larger WEBs was not successful in this investigation due to the permeability of the WEB device. A slightly reduced rescue time and fewer attempts were observed using the Microsnare when compared to the ARD; however, this difference was not statistically significant. To achieve a successful rescue using the ARD it is necessary to have direct access to the proximal marker or its surrounding area. A rotation of the WEB device during migration was not observed in this study or in the case reported by König et al. [11]. The ability of the WEB device to be centrally positioned not only within the aneurysm but also within the migrated vessel seems to be beneficial for a rescue approach using the ARD. Nevertheless, because of the rare clinical cases a rotation of a WEB device during migration cannot be ruled out. It is believed that in such cases the applied rescue approaches in this study have to be expected to be more difficult or even ineffective, especially when using the ARD, since the access to one of the markers seems to be important in order to be successful.

In summary, the most common causes of a dislocation or migration of a WEB device are retraction of the delivery wire after an incomplete detachment and coverage of the detachment zone with the microcatheter, increasing the risk of an interlocking of the catheter tip with the proximal marker of the WEB. Other factors, such as WEB oversizing and undersizing, aneurysm morphology (conical shape), elongated vascular anatomy and a complex angle between the aneurysm and the parent vessel, should also be taken into account. Considering the high thrombogenicity of the WEBs and the potential of a poor clinical outcome, it is believed that a rescue maneuver should be attempted if migration of a WEB device is encountered intraoperatively. In cases of a partial dislocation out of the aneurysm, repositioning with a microcatheter seems to be an effective alternative [11]. If repositioning with a microcatheter is not successful, of the applied rescue approaches in this study only the ARD should be considered to rescue a WEB device which is still partially within the aneurysm. Because of a high potential for complications we would not consider an aspiration or Microsnare approach in such cases.

The results of this study indicate that the aforementioned rescue approaches of migrated WEBs are fast and carry a low risk profile. When confronted with the dislocation of a small WEB device, we would choose aspiration with a large RC as a first line rescue approach, assuming that the vessel diameter allows the introduction of the RC. This may be done with one of the RCs used in the present study; however, we do not see any reason why other large RCs cannot also be used. If aspiration was not possible, we would escalate our approach to the use of either a Microsnare or an ARD. Despite the fact that we have not investigated the rescue of small WEBs using these devices in the present study, we do not see any reason why this approach should not work. In the case of migration of a larger WEB device we would use a Microsnare as a first line rescue approach, because we observed a faster rescue time with less attempts compared to the ARD.

With the growing experience and more frequent use of WEBs we believe it is important for neurointerventionalists to be aware of possible bail-out techniques in cases of migration or dislocation. Even though these complications seem to be extremely rare, it is better to know about existing bail-out techniques and not needing them than to face complications and not having an exit strategy.

### Limitations

In the porcine model used, rescue of migrated WEBs was performed in vessels representing the sizes of the MCA (M1 and M2 segment), BA and ICA in humans; however, human vessel anatomy is much more challenging in terms of tortuosity. The main limitation of the present study was that only a small number of rescue maneuvers with selected WEB device sizes could be performed. An experiment that involves all available WEB device sizes is impossible to perform. To standardize this study as effectively as possible, we kept the variation of WEBs reasonable in order to maximize statistical validity.

## Conclusion

This experimental investigation demonstrates that rescue of migrated WEB devices is a feasible and effective approach. Promising rescue rates, times and attempts have been achieved for small WEBs using reperfusion catheters (SOFIA Plus and JET 7) and for larger WEBs using dedicated rescue devices (Microsnare and ARD).

## Abbreviations

ADAPT: A direct aspiration first pass technique
ARD: Alligator retrieval device
DAPT: Dual antiplatelet therapy
DSA: Digital subtraction angiography
FPE: First Pass Effect
ICA: Internal carotid artery
MCA: Middle Cerebral Artery
RC: Reperfusion catheter
WEB: Woven Endobridge
